# Clinical relevance of targeted exome sequencing in patients with rare syndromic short stature

**DOI:** 10.1186/s13023-021-01937-8

**Published:** 2021-07-03

**Authors:** Gilyazetdinov Kamil, Ju Young Yoon, Sukdong Yoo, Chong Kun Cheon

**Affiliations:** 1 Department of Pediatrics, National Children’s Medical Center, Tashkent, Uzbekistan; 2 Division of Pediatric Endocrinology, Department of Pediatrics, Pusan National University Children’s Hospital, Yangsan, Korea; 3 Research Institute for Convergence of Biomedical Science and Technology, Pusan National University Yangsan Hospital, Yangsan, Korea

**Keywords:** Mutation, Short stature, Syndrome, Targeted exome sequencing

## Abstract

**Background:**

Large-scale genomic analyses have provided insight into the genetic complexity of short stature (SS); however, only a portion of genetic causes have been identified. In this study, we identified disease-causing mutations in a cohort of Korean patients with suspected syndromic SS by targeted exome sequencing (TES).

**Methods:**

Thirty-four patients in South Korea with suspected syndromic disorders based on abnormal growth and dysmorphic facial features, developmental delay, or accompanying anomalies were enrolled in 2018–2020 and evaluated by TES.

**Results:**

For 17 of 34 patients with suspected syndromic SS, a genetic diagnosis was obtained by TES. The mean SDS values for height, IGF-1, and IGFBP-3 for these 17 patients were − 3.27 ± 1.25, − 0.42 ± 1.15, and 0.36 ± 1.31, respectively. Most patients displayed distinct facial features (16/17) and developmental delay or intellectual disability (12/17). In 17 patients, 19 genetic variants were identified, including 13 novel heterozygous variants, associated with 15 different genetic diseases, including many inherited rare skeletal disorders and connective tissue diseases (e.g., cleidocranial dysplasia, Hajdu–Cheney syndrome, Sheldon–Hall, acromesomelic dysplasia Maroteaux type, and microcephalic osteodysplastic primordial dwarfism type II). After re-classification by clinical reassessment, including family member testing and segregation studies, 42.1% of variants were pathogenic, 42.1% were likely pathogenic variant, and 15.7% were variants of uncertain significance. Ultra-rare diseases accounted for 12 out of 15 genetic diseases (80%).

**Conclusions:**

A high positive result from genetic testing suggests that TES may be an effective diagnostic approach for patients with syndromic SS, with implications for genetic counseling. These results expand the mutation spectrum for rare genetic diseases related to SS in Korea.

**Supplementary Information:**

The online version contains supplementary material available at 10.1186/s13023-021-01937-8.

## Introduction

Height is a polygenic trait [1]; although more than 80% of height variation can be explained by genetic factors, the contribution of environmental factors is clearly established [2]. The identification of novel rare genetic causes of short stature (SS) is challenging. Syndromic SS is often associated with monogenic disorders. Over 700 common variants that explain 20% of height variation in the normal population as well as 83 rare and low-frequency coding variants explaining an additional 1.7% of variation have been reported [3, 4]. In addition, genetic variants that are outside the reach of the most statistically powered association studies [5] are considered to contribute to the missing heritability of many human traits, including common variants and rare variants of small to modest effect, or a combination of both [6].

The identification of pathogenic mutations has important implications; for example, a genetic diagnosis avoids unnecessary investigations and treatment, allows appropriate genetic counseling and the identification of comorbidities in syndromic SS, and may also lead to the earlier initiation of therapy [7]. In particular, if SS is related to dysmorphic features, a syndromic condition should be suspected and a genetic test should be conducted [8]. Exome sequencing methods, such as targeted exome sequencing (TES) and whole exome sequencing (WES), can be useful in clinical practice for the genetic diagnosis of diseases associated with SS [9, 10]. In a recent study, a potential genetic etiology in children with SS was identified in 41 patients (36%), mostly by TES/WES [11]. However, the diagnostic yield for genetic SS is highly dependent on the sequencing methods, population, availability of additional family members, and definition of a high-likelihood diagnosis [12]. In 2017, in a mutation analysis of 13 families with undiagnosed syndromic SS or overgrowth, we obtained a high diagnostic yield (46.2%) [13]. In this study, we used TES to evaluate disease-causing mutations in 34 Korean patients with suspected genetic SS and characterized the clinical features of this population. We report rare genetic diseases newly associated with SS.

## Material and methods

### Patients

This retrospective cohort study was conducted between January 2018 and January 2020. Thirty-four patients with suspected syndromic disorders based on abnormal growth (height SDS <  − 2.0 for age and sex within South Korea), and distinct facial features, developmental delay, or accompanying anomalies were recruited from Pusan National University Children’s Hospital, Yangsan, South Korea. This phenotyping was performed by one single medical geneticist and then entered into the phenotype database. This was done upon the patient’s initial presentation to the clinic before results from genetic testing had returned. Traditional genetic methods such as karyotyping, chromosomal microarray (CMA), or single-gene analysis were performed based on the initial clinical assessments in most cases, but no causative genetic defect was found. In this study, we applied TES to only the probands with undiagnosed SS. All parents except for the parents of 2 patients were available to conduct familial sanger analysis after. A total of 34 patients with SS from 33 families were enrolled.

### Collected data

The following clinical data were collected: term and birth measurements (length, weight and head circumference), postnatal growth parameters, major developmental milestones, and neurological symptoms at the time of the diagnosis. Each patient was subjected to endocrine evaluation [thyroid function test, insulin-like growth factor 1 (IGF-1) and insulin-like growth factor-binding protein 3 (IGFBP-3)], routine laboratory tests (complete blood count, erythrocyte sedimentation rate, and liver and renal function tests), and X-ray to determine bone age. Growth hormone deficiency (GHD) was defined as growth hormone peak value < 10 ng/ml on the arginine and clonidine stimulation test. We also obtained patients’ family and past history, including parental height, calculation of mid-parental height (MPH), birth history, previous medication, illness, surgery, and nutrition. For girls, MPH was (maternal height + paternal height − 13 cm)/2 and that for boys was (maternal height + paternal height + 13 cm)/2. Previous growth velocity was evaluated and body proportion was determined by measuring arm span and sitting height. The sitting height was subtracted from the patient's standing height to obtain the lower body segment value. Ultra-rare diseases were defined as disorders that occur with a prevalence of below 1 in 2,000,000 [14].

### DNA and data collection

This study was performed in accordance with the Declaration of Helsinki. Informed consent was obtained from all study participants before blood was drawn. A one‐off blood sample was collected for DNA extraction. Demographic factors and other clinical features including facial dysmorphism and accompanying body anomalies were collected from clinical records.

### Targeted exome sequencing

Pure genomic DNA was isolated from peripheral blood leukocytes using the QIAamp DNA Blood Midi Kit (Qiagen, Hilden, Germany) according to the manufacturer's protocols. The proband was evaluated using the TruSight One Sequencing Panel (Illumina, Inc., San Diego, CA, USA), which includes 125,395 probes targeting a 12 Mb region comprehensively covering the exonic regions of 4813 genes-OMIM associated genes with clinical significance. The probe size was 80-mer, targeting libraries of nearly 500 bp, enriching 350–650 bases centered symmetrically at the midpoint of the probe. The captured target regions were sequenced using the Illumina HiSeq2500 sequencer (Illumina, Inc.) according to the manufacturer’s instructions. A mean coverage of 105.49X was achieved, and 95.21% of targeted bases were read > 20 times by exome capture and sequencing. Alignments and variant calling were performed using on-instrument software, followed by filtering and customized reporting for specific genes by the analysis of imported sequence data using VariantStudio. Variants in the dbSNP135 and TIARA databases for the Korean population and variants with minor allele frequencies of > 0.5% in the 1000 Genomes database were excluded from further analyses. Only functional variants were selected as pathogenic mutations. The functional annotation tools SIFT (http://sift.jcvi.org/), PolyPhen2 (http://genetics.bwh.harvard.edu/pph2/), and MutationTaster (http://www.mutationtaster.org/) were used to evaluate novel mutations and variants of uncertain significance (VUS). Variants were classified according to the methods recommended by the American College of Medical Genetics and Genomics. Mutation nomenclature was based on the cDNA reference sequences for *RUNX2* (NM_001024630), *NOTCH2* (NM_024408.3), *SLC6A8* (NM_005629.3), *RPS6KA3* (NM_ 004586.2), *IGFALS* (NM_004970.2), *TNNT3* (NM_006757.3), *NPHP3* (NM_153240.4), *ARID1B* (NM_020732.3), *NPR2* (NM_003995.3), *KMT5B* (NM_017635.4), *CC2D1A* (NM_017721.4), *SMC3* (NM_005445.3), *MEIS2* (NM_170674.4), *PCNT* (NM_006031.5), *NOTCH2* (NM_ 024408.3), and *MED13L* (NM_015335.4).

### Validation by Sanger sequencing

Sanger sequencing was used to confirm the causative variants. All causative variants were sequenced bi-directionally using an ABI PRISM 3.1 Big Dye Terminator Kit (Applied Biosystems, Foster City, CA, USA). The sequencing products were resolved on an ABI PRISM 3130XL sequencer (Applied Biosystems) and the chromatograms were analyzed using Sequencer 4.9 (Gene Codes, Ann Arbor, MI, USA).

### Protein structural modeling

Among cases with genetically confirmed syndromic SS, protein structural modeling was performed for *NOTCH2* and *MED13L* variants. The crystal structures of the domains from wild-type *NOTCH2* and *MED13L* were generated using SWISS-MODEL [https://swissmodel.expasy.org/]. Structural images were generated using PyMOL version 29 [https://pymol.org/2/].

### Statistics

All statistical analyses were performed using the R studio software (version 3.5.1). Mann–Whitney U-test was performed for continuous variables. Fisher’s exact test was performed for categorical variables according to its characteristics. Odds ratio and confidential interval were calculated for variables.

## Results

### Clinical and genetic features of individuals with short stature

The auxological profiles, hormone results, and additional clinical features of the patients are listed in Table 1. The mean height SDS of 17 genetically diagnosed patients at the time of the hospital visit was − 3.27 ± 1.25. The mean SDS values for IGF-1 SDS and IGFBP-3 at diagnosis were − 0.42 ± 1.15 and 0.36 ± 1.31, respectively. A GHD was identified in 23.5% (4/17) of patients who underwent a GH provocation test. Two of the 17 (11.7%) patients were small for gestational age (SGA) at birth. Most patients (16/17) exhibited distinct facial features at least one or two of these, including macrocephaly or microcephaly, hypertelorism, small chin, sparse hair, triangular face, synophrys, epicanthal fold, and low-set ears. Developmental delay (DD) or intellectual disability (ID) was noted in 12 of 17 patients. Other accompanying anomalies included skeletal anomalies (9/17), microcephaly (9/17), congenital heart disease (4/17), brain anomaly (2/17), corneal opacity (1/17), and hernia (1/17).

**Table 1:** Clinical manifestations in patients with short stature

Fm No.	Pts No.	Age at dx (yrs)	Sex	Ht at dx, cm (SDS)	Wt at dx, kg (SDS)	HC at dx, cm (SDS)	MPH (cm)	GA (wks)	Birth Wt, kg (SDS)	BA	IGF-I, ng/mL (SDS)	IGF BP3, ng/mL (SDS)	Notes
**1**	**K1**	3.6	M	90.6 (− 2.07)	12.6 (− 1.90)	51.0 (0.59)	174.5	40 + 0	3.0 (− 1.26)	D	121.2 (0.20)	3298 (0.71)	Dysmorphic facial features, such as relatively macrocephaly, broad nasal bridge, deep philtrum, hypertelorism, strabismus, and low set ears, pectus excavatum, delayed teeth and closure (ossification) of fontanelle, GHD
**2**	**K2**	15.0	F	143.2 (− 3.06)	48.6 (− 0.43)	49.8 (− 3.90)	149.5	38 + 0	2.9 (− 0.32)	A	545.1 (1.92)	6530 (0.94)	Dysmorphic facial features such as hypertelorism, small mouth with dental anomalies, and low-set ears, short and broad digits/joint hyperlaxity, flexion contractures at the elbows, scoliosis, FLD, delayed language, and diabetes
**3**	**K3**	4.8	M	48 (− 2.87)	13 (− 3.33)	49.5 (− 0.99)	175.5	40 + 3	3.0 (− 1.26)	D	118.5 (− 0.17)	3600 (0.69)	Subtle dysmorphic facial features such as long face, speech delay, GHD, autistic spectrum disorder, and DD
**4**	**K4**	5.9	M	104.8 (− 3.43)	14.4 (− 4.91)	49.0 (− 1.83)	172.0	39 + 0	3.7 (0.73)	D	88.6 (− 1.31)	2280 (− 0.09)	Dysmorphic facial features such as hypertelorism, epicanthal fold, broad nose, anteverted nares, short philtrum, large mouth, thick/everted lips, and large ears, thoracolumbar spondylosis, pectus excavatum speech delay, ADHD, and ID
**5**	K5	12.0	M	133.5 (− 2.09)	36.7 (− 0.70)	N/A	166.5	40 + 0	2.8 (− 1.73)	D	66.6 (− 3.09)	514 (− 0.85)	Subtle dysmorphic facial features such as microcephaly and small mandible
**6**	**K6A**	7.8	M	111.2 (− 2.85)	17.8 (− 2.89)	51.5 (− 0.54)	167.5	34 + 6	2.2 (− 0.13)	D	166.0 (0.20)	3110 (0.23)	Dysmorphic facial features such as asymmetric face, triangular face, upward slanting palpebral fissures, Rt cryptotia, webbed neck, campto- dactyly, Rt. congenital club foot, and GHD
	**K6B**	10.9	M	128.5 (− 2.04)	30 (− 1.05)	NA		28 + 3	1.1 (0.10)	D	176.4 (− 0.16)	2980 (− 0.08)	Dysmorphic facial features such as triangular face, strabismus, inguinal and umbilical hernia, borderline ID
**7**	**K7**	19.0	M	142.7 (− 5.73)	33.1 (− 6.68)	46.0 (− 6.12)	N/A	35 + 0	2.1 (− 0.93)	D	110.4 (− 0.68)	5010 (0.56)	Dysmorphic facial features such as microcephaly, prominent and narrow nose, and micrognathia, hearing impairment, FLD, corneal opacity, CKD, diabetes, GHD, ID
**8**	**K8**	1.6	M	71 (− 3.59)	7.6 (− 3.71)	43.0 (− 3.73)	170.5	39 + 0	2.6 (− 1.74)	D	41.3 (− 0.25)	1930 (0.11)	Dysmorphic facial features, such as synophrys, highly arched eyebrows, and short nose, hirsutism, cryptorchidism, nystagmus, ASD, shortening of splenium of corpus callosum, hearing defect, and DD
**9**	**K9**	1.0	F	70 (− 2.05)	8.0 (− 1.34)	46.0 (0.74)	158.5	40 + 0	2.9 (− 1.10)	D	48.8 (− 0.29)	1930 (0.02)	Dysmorphic facial features, such as relatively macrocephaly with frontal bossing, short arms and legs, and brachydactyly
**10**	**K10**	1.0	M	63.4 (− 4.27)	6.7 (− 3.31)	41.0 (− 4.26)	N/A	26 + 2	0.9 (0.37)	D	N/A	N/A	Dysmorphic facial features, such as high forehead, plagiocephaly, hypertelorism, macrocephaly, prominent philtrum, low and posterior rotated ears, short neck, and low hairline, broad chest, pulmonary HTN, both hydroceles, ASD, DD
**11**	**K11**	9.3	F	119.7 (− 2.17)	26.1 (− 0.71)	48.5 (− 2.8)	162.0	39 + 0	3.5 (0.52)	N	165.4 (− 0.17)	4210 (0.46)	Speech and language delay, ADHD, epilepsy, and ID
**12**	**K12**	2.5	F	81.7 (− 2.30)	11.9 (− 0.85)	43.5 (− 3.00)	NA	39 + 1	2.7 (− 1.19)	D	133.1 (0.43)	3640 (3.3)	Dysmorphic facial features such as synophrys, thin downturned upper lip, and long philtrum, strabismus, TOF, scoliosis, and DD
**13**	**K13**	2	M	72.3 (− 4.10)	8.8 (− 3.56)	44 (− 3.20)	NA	37 + 1	2.46 (− 1.12)	D	52.6 (− 0.35)	1570 (− 0.13)	Dysmorphic facial features, such as low set ears, cleft palate, VSD, ectopic neurohypophysis at pituitary stalk, thinning of corpus callosum, and DD
**14**	**K14**	3	M	85.4 (− 2.80)	12.4 (− 1.40)	45 (− 2.60)	NA	42 + 0	3.4 (− 1.26)	N	87.13 (− 0.35)	2870 (2.2)	Dysmorphic facial features, such as small chin, upward nostril, and low nasal bridge, rhizomelia, genu varum, and both slipped capital femoral epiphysis
**15**	**K15**	0.8	M	60.6 (− 4.15)	7.72 (− 1.05)	42 (− 2.10)	NA	34 + 2	2.1 (− 0.38)	D	NA	NA	Dysmorphic facial features, such as coarse face, long philtrum, and inguinal hernia, hirsutism, bowing of the long bones, vertebral abnormalities, hearing defect, and DD
**16**	**K16**	9.2	F	100 (− 6.10)	15 (− 6.24)	NA		33 + 3	2.2 (0.76)	D	34.06 (− 2.25)	1260 (− 2.7)	Dysmorphic facial features, such as hypotonic open-mouth appearance, triangular face, short philtrum, hypertelorism, bulbous nasal tip, PDA, kyphoscoliosis, and ID

*A* advanced, *ADHD* attention-deficit hyperactivity disorder, *ASD* atrial septal defect, *BA* bone age, *CKD* chronic kidney disease, *D* delayed, *DD* developmental delay, *dx* diagnosis, *FLD* fatty liver disease, *Fm* family, *GA* gestational age, *GHD* growth hormone deficiency, *HC* head circumference, *Ht* height, *HTN* hypertension, *ID* intellectual disability, *Lt* left, *MPH* mid parental height, *N* normal, *NA* not available, *No* number, *PDA* patent ductus arteriosus, *Pts* patients, *Rt* right, *SDS* standard deviation score, *TOF* tetralogy of Fallot, *VSD* ventricular septal defect, *wks* weeks, *Wt* weight

The previous clinical assessments and final diagnosis for each patient are shown in Table 2. For 5 of 17 patients (K3, K5, K7, K11, and K15), it was difficult to identify a recognizable disease or syndrome based on clinical characteristics alone. These five patients were diagnosed with cerebral creatine deficiency syndrome (K3), acid-labile subunit deficiency (K5), Meckel syndrome type 7 (K7), mental retardation, autosomal recessive Type3 (K11), and Hajdu–Cheney syndrome (HCS) (K15) by TES.

**Table 2:** Genetic results for patients with short stature

Family	Previous clinical assessment	Previous conventional analysis	Final diagnosis	Gene	Nucleotide/AA change	Inheritance mode	In silico analysis			Initial classification	Re-classification	Novel	References
							SIFT	Polyphen-2	Mutation taster				
**1**	NS	*PTPN11* *SOS1*, *KRAS,* and *NRAS*	Cleidocranial dysplasia	*RUNX2*	c.578G>A/p.Arg193Gln	De novo				LPV	PV	No	[45]
**2**	TS	46,XX	Hajdu–Cheney syndrome	*NOTCH2*	c.2816C>T/p.Pro939Leu	De novo	Deleterious (0.013)	Benign (0.008)	Disease causing (0.99)	VUS	VUS	Yes	
**3**	USGD	46,XY CMA	Cerebral creatine deficiency syndrome	*SLC6A8*	c.942_944del/p.Phe315del	Het, mat				LPV	PV	No	[46]
**4**	NS	*PTPN11* *SOS1*, *KRAS,* and *NRAS*	Coffin–Lowry syndrome	*RPS6KA3*	c.1606G>T/p.Val536Phe	Het, mat	Deleterious (0.00)	Probably damaging (1.00)	Disease causing (0.99)	LPV	PV	Yes	
**5**	USGD	46,XY	Acid-labile subunit deficiency	*IGFALS*	c.1346T>G/p.Leu449Arg	Com het, pat, mat	Tolerated (0.06)	Probably damaging (0.997)	Disease causing (0.99)	VUS	LPV	Yes	
					c.1783C>T/p.Arg595Trp)		Deleterious (0.00)	Probably damaging (1.000)	Disease causing (0.99)	VUS	LPV	Yes	
**6A**	GHS	46,XY, CMA	Sheldon–Hall syndrome	*TNNT3*	c.47_49del/p.Glu18del	Het, pat				VUS	VUS	Yes	
**7**	USGD	46,XY, CMA	Meckel syndrome (Type 7)	*NPHP3*	c.73G>T/p.Gly25Cys	Het, pat Het, mat	Deleterious (0.00)	Benign (0.001) (1.00)	Disease causing (0.99)	VUS	LPV	Yes	
					c.489T>G/p.His163Gln		Tolerated (0.19)	Probably damaging (0.723)	Disease causing (0.99)	VUS	LPV	Yes	
**8**	CdLS	*NIPBL*	Coffin–Siris syndrome	*ARID1B*	c.5547delC/p.Leu1850*	De novo				PV	PV	Yes	
**9**	Achondroplasia	*FGFR3*	AMDM	*NPR2*	c.2326C>T/p.Arg776Trp	Het, pat				PV	PV	No	[29, 47]
**10**	NS	*PTPN11* *SOS1*, *KRAS,* and *NRAS*	MR, AD type51	*KMT5B*	c.2422_2425del/p.Leu808Trpfs*50	De novo				PV	PV	Yes	
**11**	USGD	46,XX, CMA	MR, AR type3	*CC2D1A*	c.1610C>T/p.Ser537Leu	Homo	Deleterious (0.03)	Probably damaging (0.997)	Disease causing (0.99)	VUS	LPV	Yes	
**12**	CdLS	*NIPBL*	CdLS type3	*SMC3*	c.1453_1455delGCT/p.Ala485del	De novo				LPV	PV	Yes	
**13**	CATCH22	CMA	MEIS2-related syndrome	*MEIS2*	c.998_1000del/p.Arg333del	De novo				PV	PV	No	[48]
**14**	Skeletal dysplasia	ND	MOPD2	*PCNT*	c.3716G>A/p.Arg1239His	Compound hetero	Tolerated (0.28)	Probably damaging (0.856)	Tolerated (0.00)	VUS	LPV	No	[49]
					c.7459C>G/p.Leu2487Val		Deleterious (0.00)	Probably damaging (0.997)	Tolerated (0.00)	VUS	LPV	No	[50]
**15**	USGD	46,XY MLPA	Hajdu–Cheney syndrome	*NOTCH2*	c.5471G>A/p.Arg1824His	De novo	Deleterious (0.001)	Benign (0.008)	Disease causing (0.99)	VUS	VUS	Yes	
**16**	CATCH22	CMA	MED13L syndrome	*MED13L*	c.5444C>T/p.Thr1815Met	Het, mat	Tolerated (0.17)	Probably damaging (1.000)	Disease causing (0.99)	VUS	LPV	Yes	

*AA* amino acid, *AD* autosomal dominant, *AMDM* acromesomelic dysplasia Maroteaux-type, *AR* Autosomal recessive, *CdLS* Cornelia de Lange syndrome, *CMA* chromosomal microarray, *GHS* Goldenhar syndrome, *hemi* hemizygote, *het* heterozygote, *LPV* likely pathogenic variant, *mat* maternal origin, *MOPD2* microcephalic osteodysplastic primordial dwarfism type II, *MR* mental retardation, *N/A* not available, *ND* not done, *NS* Noonan syndrome, *pat* paternal origin, *Pts* patients, *PV* pathogenic variant, *Ref* references, *TS* Turner syndrome, *USGD* unrecognizable syndromic growth disorder, *VUS* variant of uncertain significance

Three (17.6%) patients (K1, K4, and K10) had suspected syndromes, such as Noonan syndrome, based on facial and clinical features, but no mutations were identified by Sanger sequencing of *PTPN11*, *SOS1*, *RAF1*, and *KRAS*. Patient K1 showed dysmorphic facial features, such as relative macrocephaly, broad nasal bridge, deep philtrum, hypertelorism, low set ears, strabismus, pectus excavatum, delayed teeth and closure (ossification) of the fontanelle, and GHD. The patient was confirmed to have cleidocranial dysplasia with a de novo missense mutation, c.578G>A (p.R193Q), in *RUNX2*. Patient K4 displayed dysmorphic facial features, such as hypertelorism, epicanthal fold, broad nose, anteverted nares, short philtrum, large mouth, thick/everted lips, large ears, thoracolumbar spondylosis, pectus excavatum, speech delay, and severe ID. A novel hemizygous missense variant, c.1606G>T (p.V536F), in *RPS6KA3* was identified in a patient with Coffin–Lowry syndrome. Patient K10 displayed severe SS (− 4.27 SDS). He showed dysmorphic facial features, such as a high forehead, plagiocephaly, hypertelorism, macrocephaly, prominent philtrum, low and posterior rotated ears, short neck, low hairline, round shoulder, broad chest, congenital heart defect, and DD. A genetic analysis revealed a de novo novel frameshift mutation (p.L808Wfs*50) in *KMT5B*, resulting in mental retardation, autosomal dominant type 51.

**Fig. 1:**
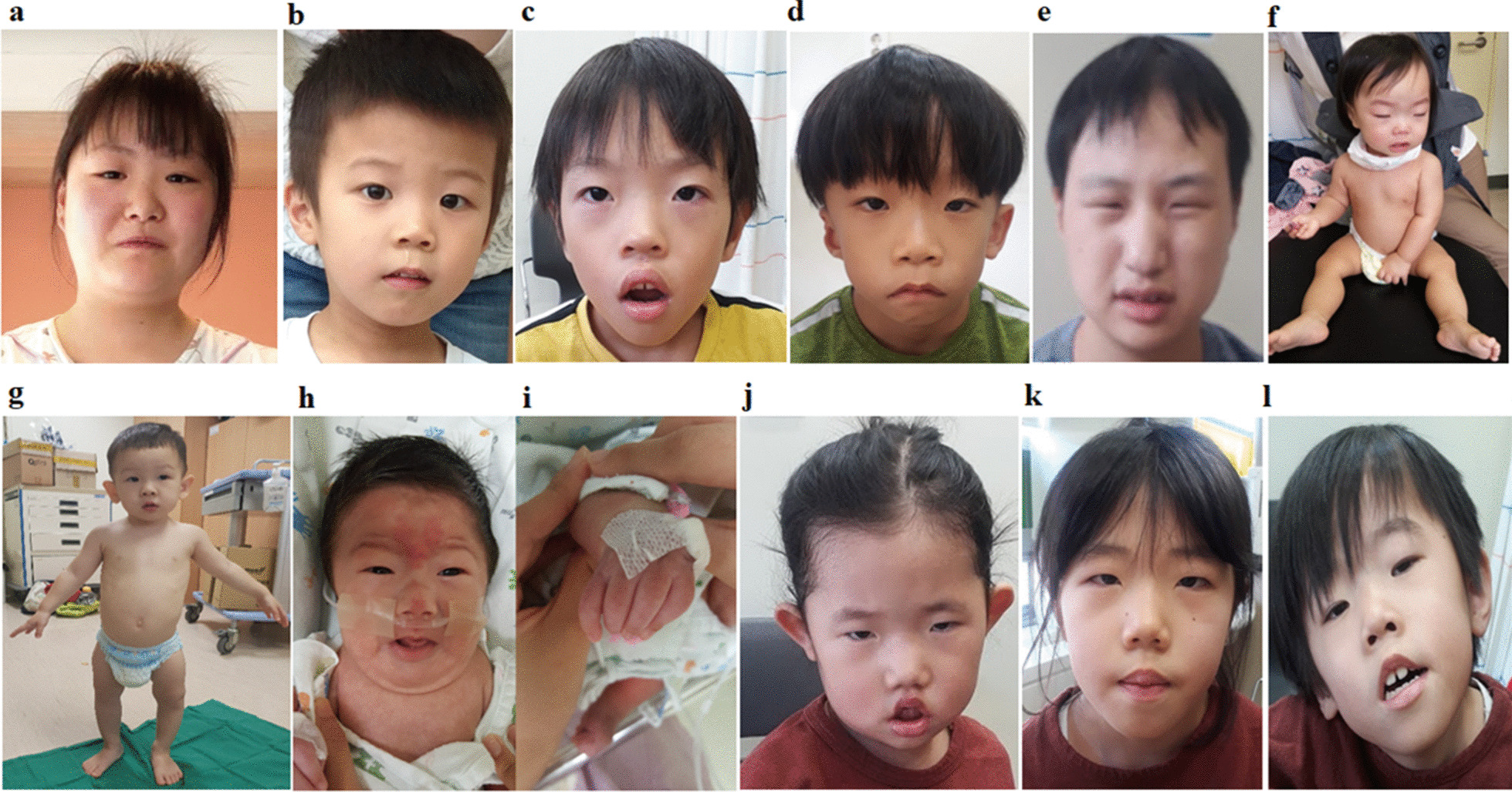
Characteristic features of the face and body in individuals with short stature. **a** Hypertelorism, small mouth, and low-set ears, in patient K2 with Hajdu–Cheney syndrome; **b** subtle dysmorphic facial features, such as long face, in patient K3 with cerebral creatinine deficiency; **c** hypertelorism, epicanthal fold, broad nose, anteverted nares, short philtrum, large mouth, thick/everted lips, and large ears, in patient K4 with Coffin–Lowry syndrome; **d** triangular face, upward slanting palpebral fissures, right cryptotia, nasolabial folds, small mouth with a high, arched roof of the mouth, and webbed neck in patient K6 with Sheldon–Hall syndrome; **e** microcephaly, prominent and narrow nose, short philtrum, and micrognathia in patient K7 with Meckel syndrome (Type 7); **f** relative macrocephaly with frontal bossing, short arms and legs, and brachydactyly in patient K9 with acromesomelic dysplasia Maroteaux-type; **g** small chin, upward nostril, and low nasal bridge, rhizomelia, and genu varum in patient K14 with microcephalic osteodysplastic primordial dwarfism type II; **h**, **i** coarse face, long philtrum, and hand deformity in patient K15 with Hajdu–Cheney syndrome; **j** hypotonic fish-mouth appearance, hypertelorism, and flat nasal bridge with bulbous nasal tip in patient K16 with MED13L syndrome; **k**, **l** her (patient K16) siblings with the same mutation in *MED13L*

**Fig. 2:**
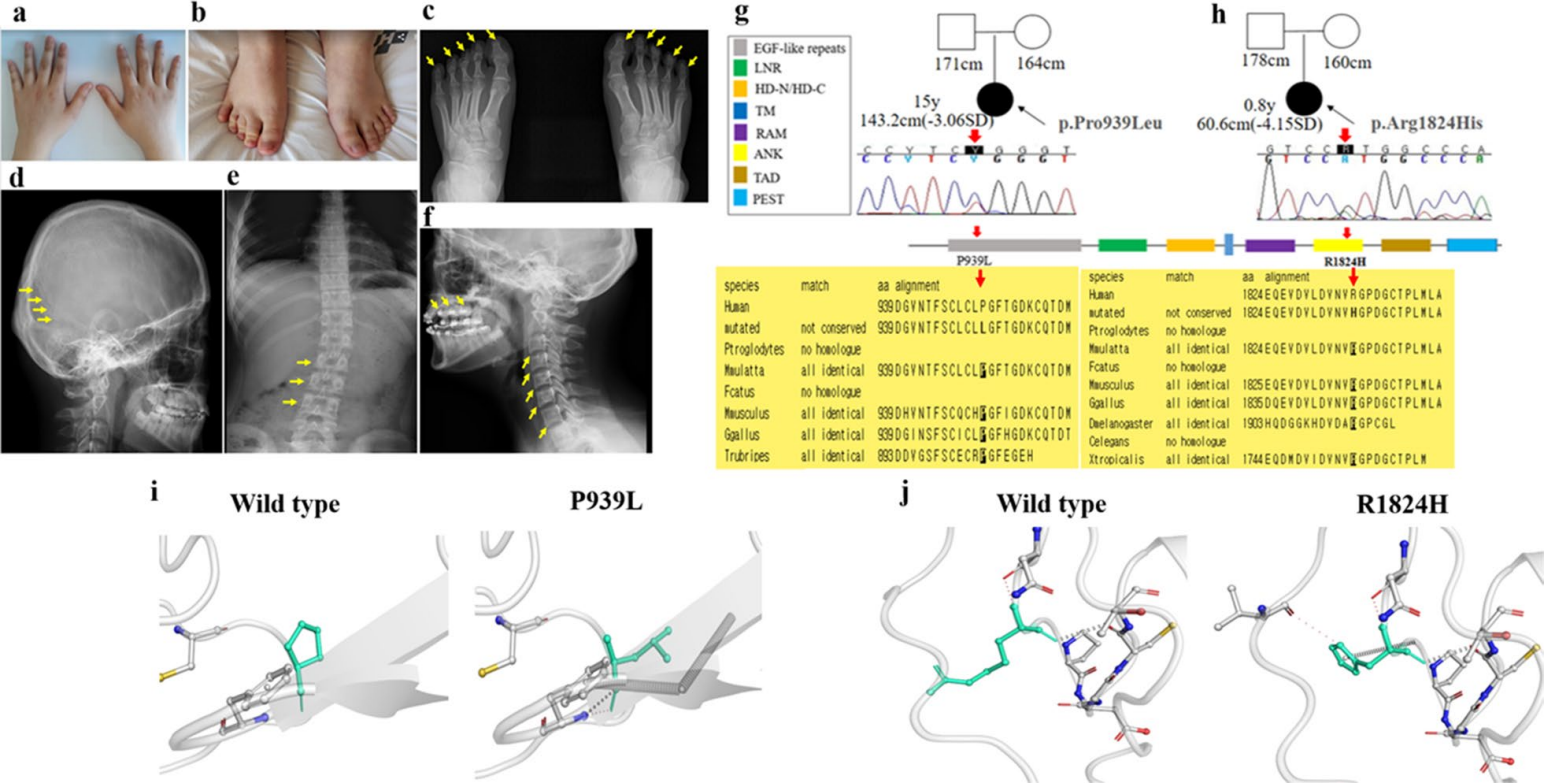
Visual findings, radiographic skeletal survey, and genetic analysis in patient K2 with Hajdu–Cheney syndrome. **a**, **b** Short and broad digits were observed; **c** cone-shaped distal phalanges due to peripheral acrolysis is indicated by yellow arrows; **d** wormian bones are marked by yellow arrows in the lambdoidal suture; **e** lumbar scoliosis is marked by yellow arrows; **f** malocclusion and biconcave vertebra bodies marked by yellow arrows. Two novel heterozygous variants in the *NOTCH2* gene (NM_024408.3). **g**, **h** Sanger sequencing confirmed two de novo novel variants, c.2816C>T (p.P939L) and c.5471G>A (p.R1824H), in *NOTCH2* in patients K2 and K15, respectively, shown by the red arrow. P939 located on EGF-like repeats and R1824H on ankyrin repeat domain residues are conserved among species, shown in the dark square box. Sequences were aligned using blastp [https://blast.ncbi.nlm.nih.gov/]; **i**, **j** wild-type and mutant residues (p.P939L and p.R1824H) in the NOTCH2 protein are shown in light-green and are also represented as sticks alongside the surrounding residues, indicating any type of interaction. Purple dots represent metal complex interactions with a surrounding residue. Orange dots represent weak hydrogen bonds with a surrounding residue. The crystal structure of the domain from wild-type NOTCH2 was generated by SWISS-MODEL [https://swissmodel.expasy.org/] and depicted as a cartoon representation. All structural images were generated using PyMOL (https://pymol.org/)

Patient K2 was initially suspected of having Turner syndrome with a severe SS (− 3.06 SDS), dysmorphic facial features, such as low set ears, short and broad digits, and cubitus valgus (Figs. 1a, 2a, b). Her skeletal survey revealed wormian bones in the lambdoidal suture, scoliosis, malocclusion, biconcave vertebra body, and cone-shaped distal phalanges due to peripheral acro-osteolysis (Fig. 2c–f). Finally, she was diagnosed with HCS caused by a de novo missense variant, c.2816C>T (p.P939L) in the *NOTCH2* gene (Fig. 2g).

Patient K6 was initially suspected of having Goldenhar syndrome based on clinical manifestations, such as asymmetric face, right cryptotia, nasolabial fold, and small mouth with a high arched roof of the mouth (Fig. 1d). He displayed camptodactyly and a congenital club foot. He had GHD with a delayed bone age. A novel small deletion, c.47_49del (p.E18del), in *TNNT3* was identified in the proband and his family members and was responsible for Sheldon Hall syndrome (SHS).

Two (11.7%) patients (K8 and K12) were initially suspected of having Cornelia de Lange syndrome based on dysmorphic facial features, such as synophrys and highly arched eyebrows, but mutations in *NIPBL* were not detected. Patient K8 was ultimately diagnosed with Coffin–Siris syndrome with a de novo novel variant, c.5547delC (p.L1850*), in *ARID1B* and patient K12 harbored a de novo novel variant, c.1453_1455delGCT (p.A485del), in *SMC3*, which is related to a mild variant of Cornelia de Lange syndrome with predominant ID.

**Fig. 3:**
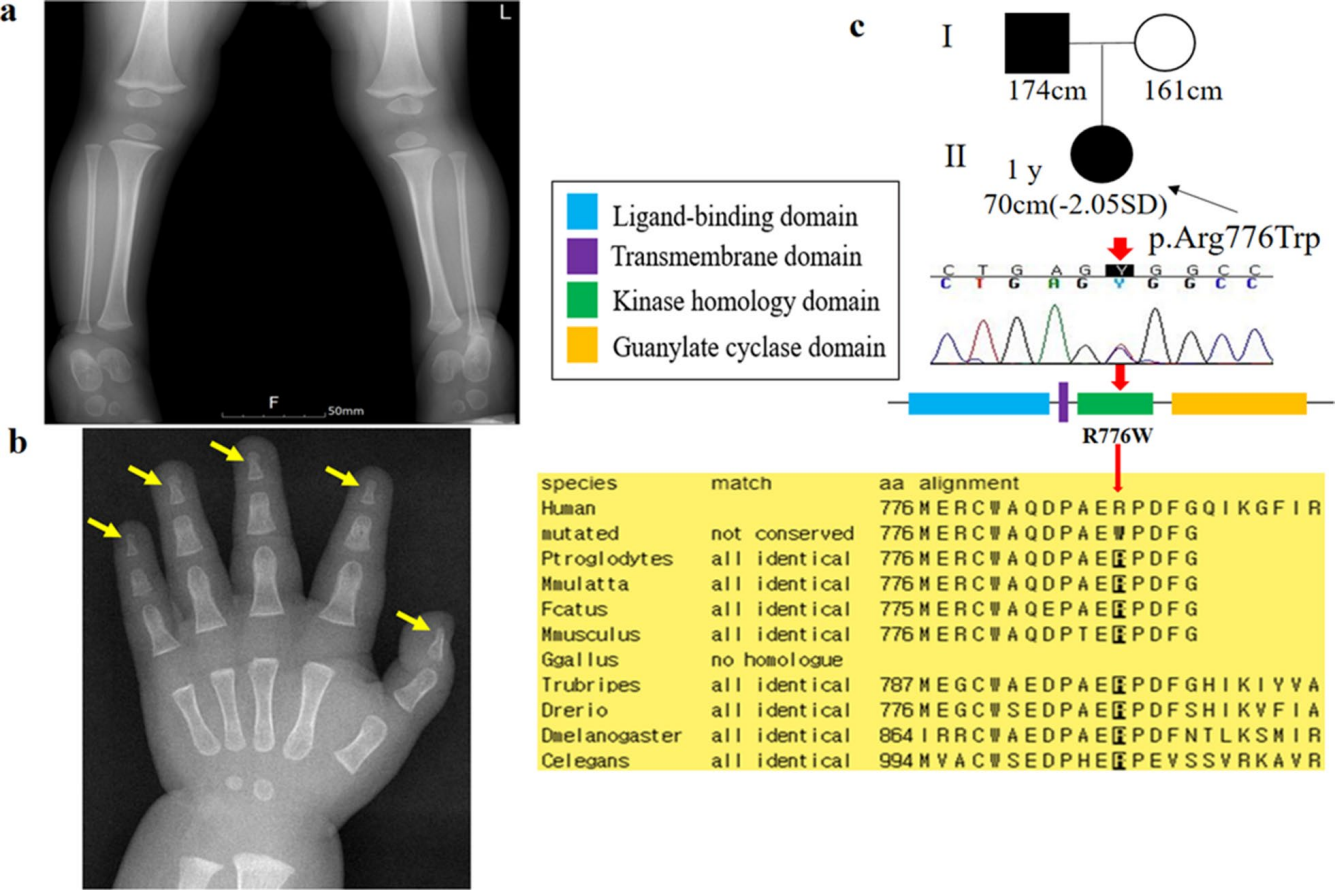
Radiographic image and molecular analysis of patient K9 with acromesomelic dysplasia Maroteaux-type. **a** Lower extremities X-rays demonstrated a short tibia and fibula, with no missing or fused bones; **b** phalanges of the hands were short and broad with cone-shaped epiphyses, especially in the 4th proximal phalange marked by yellow arrows; **c** Sanger sequencing confirmed a missense mutation, c.2326C>T (p.A776W) (NM_003995.3), in the kinase homology domain of the *NPR2* gene in the proband and her father. The A776 residue is conserved among species (dark square box). Sequences were aligned using blastp [https://blast.ncbi.nlm.nih.gov/]

Patient K9 was initially presumed to have achondroplasia during infancy; however, no mutation was found in *FGFR3*. Dysmorphic facial features, such as relatively macrocephaly with frontal bossing and micrognathia, were observed (Fig. 1f). Her height was 70 cm (− 2.05 SDS), with markedly short upper and lower extremities. Lower body X-rays demonstrated short tibia and fibula, with no missing or fused bones (Fig. 3a). Phalanges of the hands were short and broad with cone-shaped epiphyses, especially in the 4th proximal phalange (Fig. 3b). A missense mutation, c.2326C>T (p.A776W), in the kinase homology domain of the *NPR2* gene was identified in the proband and her father (Fig. 3c).

Patient K14 displayed SS (− 2.8 SDS), genu varum, rhizomelia, and facial dysmorphism, including small chin, upward nostril, and low nasal bridge (Fig. 1g). His father was Korean and his mother was Japanese. Initially, he was suspected to have skeletal dysplasia. TES revealed a compound heterozygous mutation, c.3716G>A (p.R1239H) and c.7459C>G (p.L2487V), in *PCNT* responsible for microcephalic osteodysplastic primordial dwarfism (MOPD) type II, inherited from his parents.

**Fig. 4:**
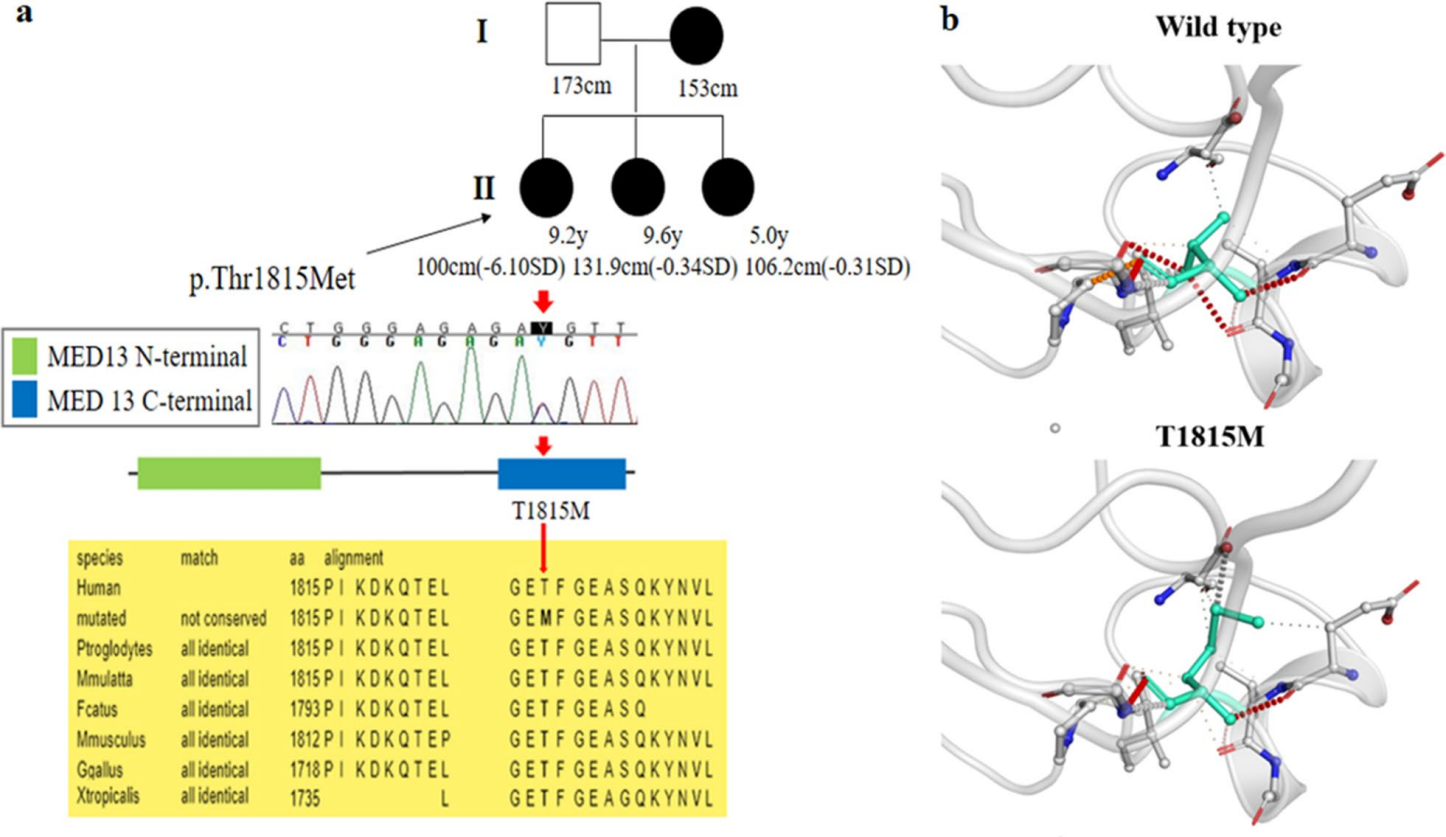
Pedigree and molecular and protein structural analyses of patient K16 with MED13L syndrome. **a** A novel missense variant, c.5444C>T (p.T1815M) (NM_015335.4), in the *MED13L* gene, was identified in the mother and three daughters. T1815M is located on the mediator complex subunit 13 C-terminus and is highly conserved across species. Sequences were aligned using blastp [https://blast.ncbi.nlm.nih.gov/]; **b** wild-type and mutant residues (T185M) in the MED13L protein are shown in light green and are also represented as sticks alongside the surrounding residues, indicating any type of interaction. Red dots represent hydrogen bonds, orange dots indicate weak hydrogen bonds, and green dots hydrophobic contacts. Protein crystallization predicted that the T1815M variant on the surface of the protein affects hydrogen bonds and hydrophobic interactions with a nearby residue to decrease the stability of an alpha-helix. The crystal structure of the domain from wild-type MED13L was generated using SWISS-MODEL [https://swissmodel.expasy.org/] and depicted as a cartoon representation. All structural images were generated using PyMOL (https://pymol.org/)

Two (11.7%) patients (K13 and K16) were initially suspected to have CATCH22 syndrome based on facial features, but no microdeletion was detected by chromosomal microarray. Patient K13 displayed severe SS (− 4.10 SDS), dysmorphic facial features, such as low set ears, cleft palate, and congenital heart defect. A de novo small deletion (c.998_1000del) in *MEIS2* responsible for MEIS2-related syndrome was detected. Patient K16 presented with proportionate SS (− 6.10 SDS) and ID. She had facial dymorphism, such as fished mouth and hypertelorism (Fig. 1j), congenital heart disease, and hydrocephalus. Her mother and two siblings also had ID and similar dysmorphic facial features, such as a fish-mouth appearance, flat nasal bridge with bulbous nasal tip, hypertelorism, and triangular face. A novel missense mutation, c.5444C>T (p.T1815M), in *MED13L* associated with MED13L syndrome was identified in the proband and her siblings, and this mutation was inherited from her mother (Fig. 4a).

The comparison of clinical features between genetically positive cases and negative cases are depicted in Additional file 1. The frequency of the dysmorphic face was statistically higher in the positive cases compared to the negative cases (94.1% vs. 52.9%, *p* = 0.017, odds ratio 13.15).

### Diagnostic yield, classification, and characteristics of variants

**Fig. 5:**
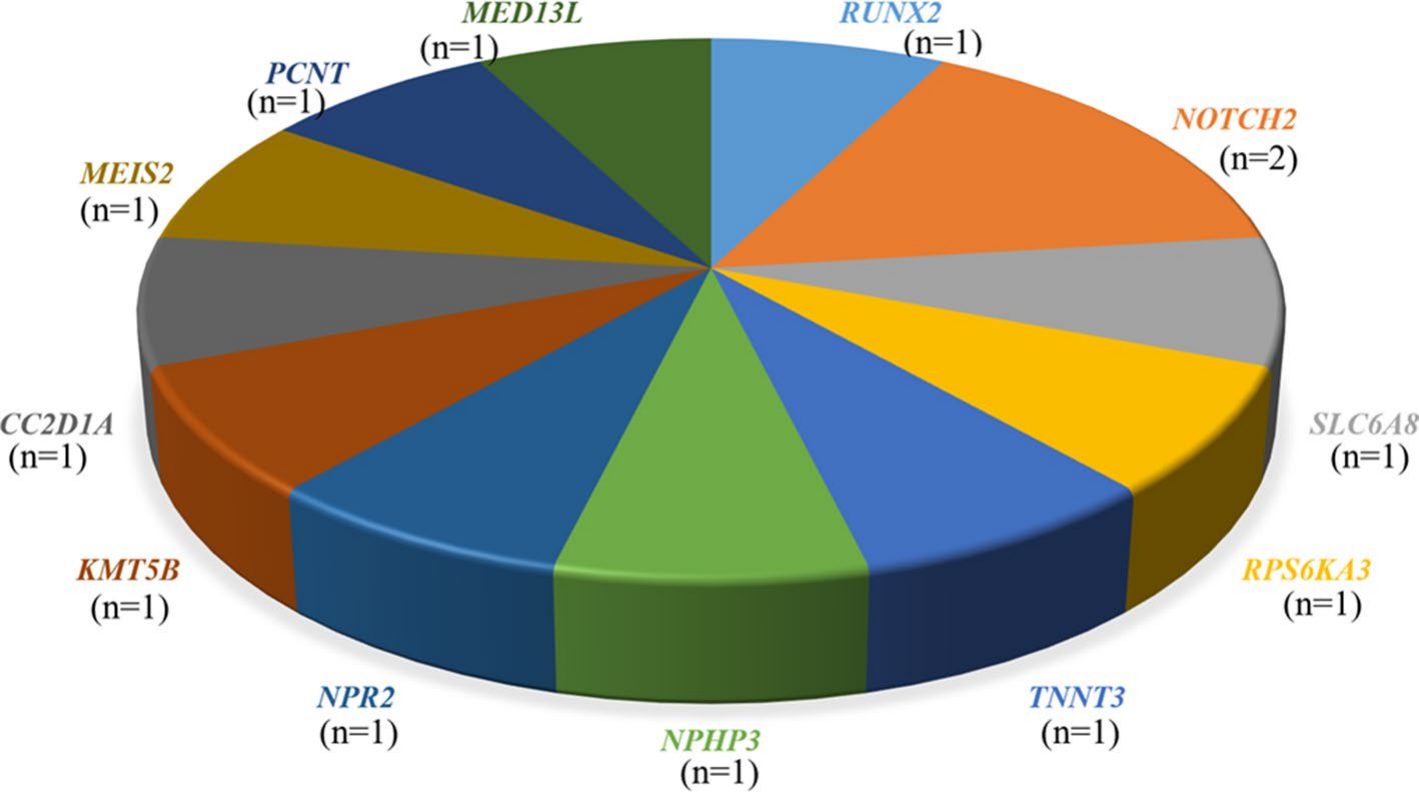
Mutated genes related to ultra-rare diseases identified in the study

Among 34 patients with suspected syndromic SS, genetic diseases were identified in 17 patients (50%) when PV, LPV, and VUS are included. However, when only PV and LPV excluding VUS are included, the diagnosis rate was 38.2%. Sixteen (94.1%) underwent a genetic and/or cytological analysis prior to TES. These patients were assessed by Sanger sequencing as well as family member testing and segregation analyses, yielding 19 variants, including 13 (68.4%) novel variants responsible for 15 genetic disorders (Table 2). Nine novel missense variants were predicted to be deleterious by at least one of three bioinformatics algorithms (i.e., SIFT, PolyPhen2, and MutationTaster). With respect to the mode of inheritance, 40% cases were autosomal dominant de novo (6/15), 26.7% were autosomal recessive (4/15), 13.3% were X-linked (2/15), and 13.3% were autosomal dominant with unknown inheritance (2/15). Looking at the distribution of causative variants, pathogenic variants accounted for 21.1% (4/19), likely pathogenic variants accounted for 21.1% (4/19), and VUS accounted for 57.9% (11/19) (Table 2). However, after re-classification by clinical reassessment, such as family member testing and segregation studies, pathogenic variants accounted for 42.1% (8/19), likely pathogenic variants 42.1% (8/19), and VUS 15.7% (3/19) (Table 2). For confirmed causative variants, missense was the most common type, with a frequency of 68.4% (13/19), followed by frame-in/del with a frequency of 26.3% (5/19) and frameshift with a frequency of 5.3% (1/19). Ultra-rare diseases accounted for 12 out of 15 genetic diseases (80%) (Fig. 5).

### Identification of ultra-rare genetic diseases and its impact on clinical management

**Fig. 6:**
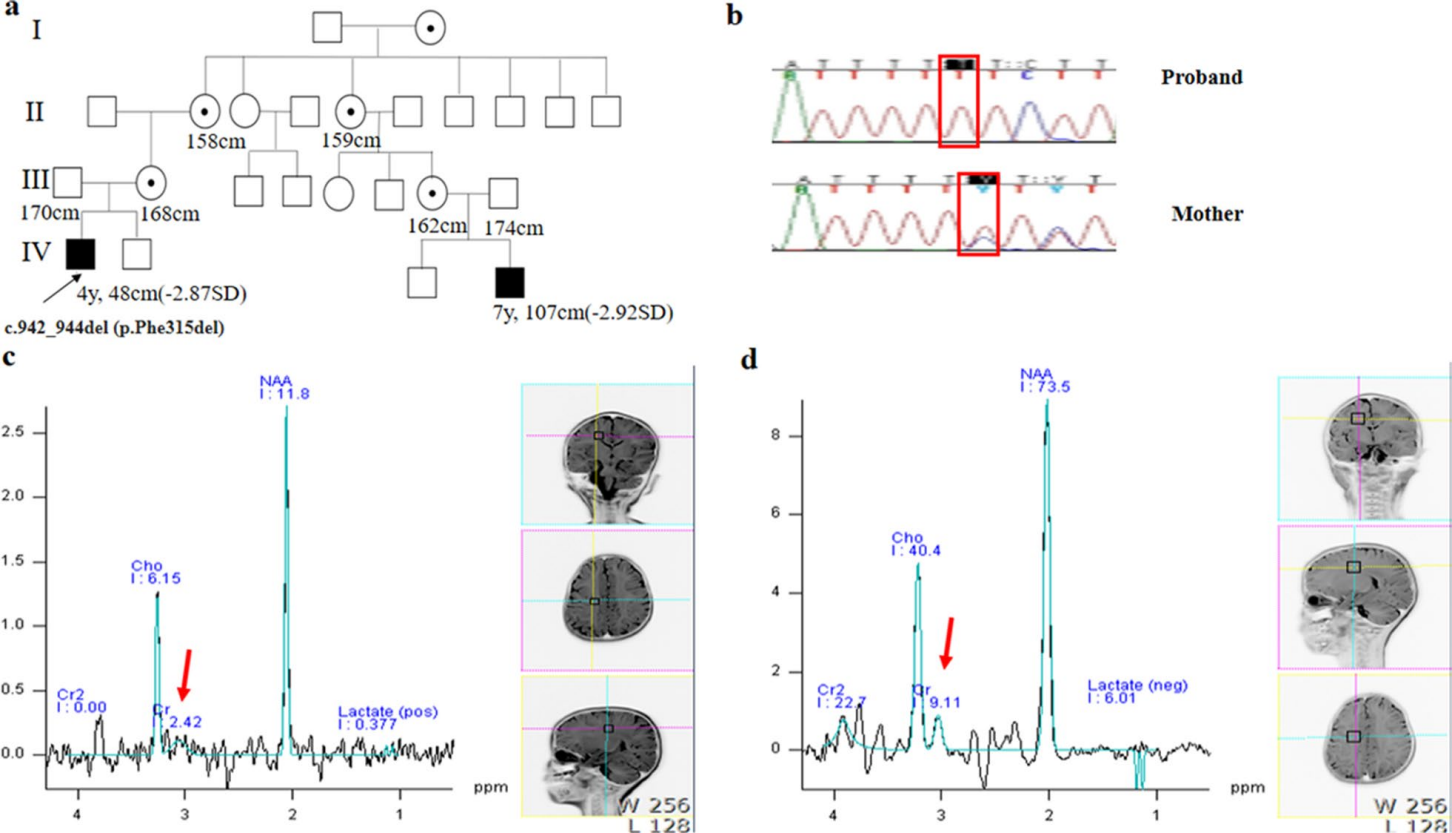
Pedigree and molecular analysis and brain image work-up for patient K3 with creatinine cerebral deficiency. **a** A pedigree analysis shows that the genetic disease in the family of patient 2 is inherited in an X-linked recessive manner; **b** Sanger sequencing confirmed a hemizygous in-frame deletion variant, c.942_944del (p.F315del) (NM_005629.3), in *SLC6A8* identified by targeted exome sequencing, shown in the red rectangular box; **c** MR spectroscopy reveals a diminished creatine signal peak, indicated by thick red arrows in both the parietal lobe and right temporal lobe at baseline. Concentration of *N*-acetyl aspartate (NAA) and cholines (Cho) were within normal ranges; **d** MR spectroscopy demonstrates an elevated creatine signal peak indicated by the thick red arrow in both the parietal lobe and right temporal lobe after 2.5 years of oral treatment with creatine, arginine, and glycine supplementation

The confirmation of an ultra-rare genetic disease by TES affected patient management. Disease monitoring was initiated in 52.9% of patients. In addition, the systemic involvement of specific genetic diseases was investigated in 41.1% of patients, the estimated inheritance pattern was changed in 11.7%, and prognosis was changed in 11.7%. Medical interventions were initiated in 5.8% of patients, and reproductive planning was initiated in 5.8% of patients. For example, patient K3 displayed a speech delay, suspected autism spectrum disorder, and SS. He had only been receiving speech rehabilitation treatment for unknown speech delays. CMA and karyotyping analyses showed negative results. After TES, a hemizygous small deletion, c.942_944 (p.F315del), in *SLC6A8,* responsible for X-linked cerebral creatine deficiency, was detected in both the patient and his mother (Fig. 6a, b). His mother's cousin's son also showed speech delays and SS. Baseline MR spectroscopy showed a decreased creatine peak level of 2.42 ppm in both the parietal and right temporal lobes (Fig. 6c), and a urine analysis revealed a significant elevation in the creatine/creatinine ratio (43.9, ref; 0.017–0.720) corresponding to a creatine transporter deficiency. After the final diagnosis, the patient was administered oral arginine (400 mg/kg/day), glycine (150 mg/kg/day), and creatine (400 mg/kg/day). We observed progressive improvements in developmental skills, such as the Sequenced Language Scale for Infants (from < 1% percentile to 3% percentile), K-Vineland-II (SQ from 37 to 40, K-CARS-ST (from 38.5 to 35), and K-CARS-2 (from 100 to 65), as well as an increased creatine peak of 9.11 ppm in both the parietal and right temporal lobes by MR spectroscopy (Fig. 6d) and a decreased creatine/creatinine ratio (43.9, ref; 0.017–0.720) after 2.5 years of oral supplementation.

## Discussion

In a study of 34 patients with clinically suspected syndromic SS, we identified 19 genetic variants in 17 (50%) patients, including 13 (68.4%) novel variants responsible for 15 rare genetic disorders.

Originally, in 6 of 17 patients (35.2%), the cause of SS was generally considered to be GHD or SGA and was finally identified as an underlying genetic disease by TES. In clinical practice, in-depth TES analyses can reveal various genetic diseases related to SS, including ultra-rare diseases, emphasizing the importance of this approach in pediatric endocrinology. A cohort study of Chinese children with SS showed a high diagnostic yield of 33.3% through TES/WES comparing to that of chromosomal microarray analysis (1.8%) [15]. Similarly, a previous smaller study of 14 highly selected patients with SS reported a diagnostic yield of 36% through WES [16]. However, Hauer et al. reported a slightly lower diagnosis rate of 16% through WES to identify the underlying cause of SS [17]. On the other hand, Freire et al. reported a diagnostic yield of 15% through target panel and exome sequencing [18]. The diagnostic yield might be affected by the study’s strict inclusion criteria, test methods, and sample size.

In this study, if we look closely at the rare monogenic causes of SS identified through TES, two patients (11.7%) were diagnosed with HCS, a rare connective tissue disease characterized by acroosteolysis of the hands and feet, developmental defects of bones and joints causing distinctive craniofacial and skull changes, as well as severe osteoporosis and SS [19]. The craniofacial developmental abnormalities are probably related to the effects of Notch on skeletal development, and the SS may be secondary to the inhibitory effects of Notch on chondrogenesis [19]. Based on the limited number of HCS cases reported to date, the common pathogenic mechanism seems to involve nonsense mutations or deletions, leading to a termination codon in exon 34 of *NOTCH2* upstream of the PEST domain [20–22]. Interestingly, two de novo novel variants, P939L and R1824H, in two cases (K2 and K15) in the present study were located in the calcium-binding EGF-like domain and Ankyrin 2 domain, respectively (Fig. 2g, h), which has not been reported previously. The allele frequencies of these two variants in East Asia were 0.001403 and 0.000000, respectively. Protein crystal structures revealed that the P939L variant might affect the metal complex interactions with surrounding residues (Fig. 2i), while the R1824H variant affected weak hydrogen bonds and metal complex interactions (Fig. 2j).

Rare skeletal disorders leading to severe SS and abnormal skeletal morphologies often have genetic etiologies in children. In this study, rare genetic skeletal disorders were identified by TES. First, SHS (MIM# 601680) or distal arthrogryposis type 2B is an autosomal dominant disorder characterized by congenital contractures of the distal joints of the limbs without a primary neurological defect [11]. The most common clinical features of SHS include a triangular face, prominent nasolabial folds, small mouth, high arched palate, attached earlobes, mild cervical webbing, SS, severe camptodactyly, ulnar deviation, and vertical talus and/or talipes equinovarus [11]. In the present study, the first Korean family with a novel variant in *TNNT3* associated with SHS was identified in patient K6. Although SHS is a rare disease, it can exhibit autosomal dominance, and familial recurrence has been reported in approximately 50% of cases [13]. Our patient had metatarsus varus at a young age. His grandmother, father, uncle, and elder brother were all short. Interestingly, while his intelligence was normal, his uncle and brother showed a mild ID, suggesting phenotypic variability. Recently, mutations in genes encoding the skeletal muscle contractile fiber complex (*TNNI2*, *TNNT3*, *TPM2*, *MYH3,* and *MYBPC1*) have been identified as causes of distal arthrogryposis types 1 and 2 [23]. *MYH3* mutations account for 32% of SHS cases [24]. In Korea, only patients with mutations in *TPM2* related to SHS have been reported previously [25]. For SHS with genetic heterogeneity corresponding to phenotypic variation, TES can be a very useful diagnostic tool.

Second, acromesomelic dysplasia Maroteaux-type (AMDM) was detected in patient K9 with heterozygous missense mutation, R776W, in *NPR2*. AMDM is a rare autosomal recessive skeletal disorder that leads to severe SS and an abnormal skeletal morphology [26]. The height of her father, who was a carrier with AMDM, was normal (174 cm), and he had the normal shape and proportion of the skeletal elements. Individuals with classic AMDM have severe SS with significant shortening of the middle and distal parts of their limbs, especially the hands and feet [27]. Interestingly, carriers with AMDM often have SS and mild skeletal defects; however, height in *NPR2* heterozygotes is highly variable, ranging from short to normal [26, 28]. The R776W mutation located within the kinase homology domain was originally reported in a Turkish family, and carriers of this mutation are shorter than matched controls [29]. The kinase homology domain binds to ATP and contributes to the regulation of guanylyl cyclase activity and bone growth in response to C-type natriuretic peptide [30]. Bartels et al. found that the average height of 30 adult carriers of AMDM was 5.7 cm shorter than that of population-matched controls [29]. Recently, Hwang et al. identified heterozygous *NPR2* mutations in 2.6% of Korean subjects with idiopathic SS, and these mutations have a dominant-negative effect on growth signals based on in vitro functional analyses [31].

Third, MOPD type II was identified in patient K14; this condition is characterized by the disproportionate shortening of the fore-arms and legs, short stature with other skeletal abnormalities (osteodysplasia), microcephaly, and a phenotype consistent with Seckel syndrome [32]. Other skeletal abnormalities include abnormal development of the hip joints (hip dysplasia), cox vara, thinning of the bones in the arms and legs, and shortened wrist bones [32]. Our patient also displayed cox vara, genu varum, and both slipped capital femoral epiphysis, compatible with MOPD type II (Additional file 2). The pericentrin gene (*PCNT*) [OMIM #605925] encodes a large coiled-coil protein that localizes to the pericentriolar material, where it interacts with several structural centrosomal proteins, including γ-tubulin and PCM1, involved in centrosome function and spindle assembly [33]. The perturbation of cell division not only affects brain size but also body size [34]. Of interest, most pathogenic mutations previously reported in patients with MOPD type II are truncation mutations [34, 35], whereas our patient had two missense mutations, c.3716G>A (R1239H) and c.7459C>G (L2487V), in *PCNT*. The R1239H variant was located on the structural maintenance of chromosomes (SMC) protein domain, while L2487V is expected to interact with NEK2, which plays a key role in the regulation of mitotic processes.

In addition to the skeletal disorders causing SS, we observed a rare genetic syndrome, MED13L, which involves abnormalities in both early heart and brain development [36]. Individuals with MED13L syndrome develop postnatal SS [37]. The common facial gestalt shows some resemblance to that of 22q11.2 microdeletion syndrome [38]. The majority of reported mutations are de novo, and missense mutations remain VUS [32]. Patient K16 was the first Korean familial case with a novel missense variant, c.5444C>T (p.T1815M), in *MED13L*, which is maternally inherited. This variant was not found in the ExAC or 1000 Genomes databases. T1815M is located on the mediator complex subunit 13 C-terminus and is highly conserved across species (Fig. 4a). Protein crystal structures predicted that the T1815M variant located on the surface affects hydrogen bonds and hydrophobic interactions with a nearby residue to decrease the stability of an alpha-helix, thereby affecting the MED13L secondary structure (Fig. 4b). MED13L, a protein with an uncharacterized structure, is a subunit of the mediator complex that links DNA-binding transcription factors and RNA polymerase II for gene transcription [39]. The mediator is an evolutionarily conserved multi-protein complex with diverse and dynamic roles at multiple stages of transcription [40]. To date, 26 patients with likely truncating/loss-of-function de novo aberrations of *MED13L* have been reported, all showing ID/DD and a spectrum of facial anomalies [38]. Notably, the older sister and younger sister of the patient carried the same mutation, and both had similar facial appearances and ID without SS (− 0.34 SDS and − 0.31 SDS, respectively). The mother showed similar facial appearances to those of her daughters but showed a normal height (− 1.25 SDS) and intellectual functions. The phenotype variability in a Korean family with MED13L syndrome may be associated with a novel *MED13L* variant.

Finally, as a result of comparing the clinical characteristics such as height SDS, facial dysmorphism, visual involvement, intellectual disability or developmental delay, microcephaly, skeletal deformities, congenital heart disease, and renal anomalies of the genetically positive group and the negative group, the frequency of facial dysmorphism was higher in the genetically positive group than in the genetically negative group. Therefore, if the child short stature and facial dysmorphism is accompanied, it is expected that the possibility of identifying the underlying etiology of short stature may be high when the genetic evaluation using TES is performed.

However, this study has diagnostic limitations. Of the 19 alleles found in 17 patients, 3 (15.7%) were identified as VUS, limiting their diagnostic value. Therefore, it will be necessary for the future to verify the pathogenicity of variants by various algorithmic-based methods and functional studies to improve the robust diagnosis rate. In addition, no pathogenic variants were found in the remaining 17 patients. It is required to perform a further genetic work-up through long-read trio sequencing among individuals with unsolved cases. Because short-read sequencing methods often lack sensitivity and specificity for a large proportion of structural variants (SV) due to technical limitations [41, 42]. Each human genome harbors thousands of SV, in total spanning more than 10 Mb, that have largely remained undetected with conventional short-read sequencing [41–44]. These limitations can be overcome by long-read sequencing.

In conclusion, a positive result (50.0%) from genetic testing was high in patients with suspected syndromic SS, including the identification of 13 novel heterozygous variants by TES. These results support the use of TES as a promising tool for uncovering the causes of syndromic SS and broadly contribute to our understanding of the etiology and clinical features of SS in Korea.

## Supplementary Information

**Additional file 1.** **Supplementary 1.** Comparison of clinical features between genetically positive cases and negative cases.

**Additional file 2.** Radiographic image of patient K14 with microcephalic osteodysplastic primordial dwarfism (MOPD) type II. X-ray findings show coxa vara, genu varum, and both slipped capital femoral epiphysis marked by yellow arrows.

## Data Availability

The datasets used and/or analyzed during the current study are available from the corresponding author on reasonable request.

## Abbreviations

TES: Targeted exome sequencing
WES: Whole exome sequencing
CMA: Chromosomal microarray
SS: Short stature
MPH: Mid-parental height
SDS: Standard deviation score
IGF-1: Insulin-like growth factor 1
IGFBP-3: Insulin-like growth factor-binding protein 3
HCS: Hajdu–Cheney syndrome
GHD: Growth hormone deficiency
MOPD: Microcephalic osteodysplastic primordial dwarfism
SHS: Sheldon Hall syndrome
AMDM: Acromesomelic dysplasia Maroteaux-type
VUS: Variant of uncertain significance
