# Supplementary material for: Clinical relevance of targeted exome sequencing in patients with rare syndromic short stature
Source: Orphanet J Rare Dis. 2021 Jul 3;16:297. doi: 10.1186/s13023-021-01937-8 (PMC8254301; doi:10.1186/s13023-021-01937-8)
Supplement: Supplementary file 1 — Additional file 1. Supplementary 1. Comparison of clinical features between genetically positive cases and negative cases. [file 13023_2021_1937_MOESM1_ESM.docx]

**Supplementary 1 Comparison of clinical features between genetically positive cases and negative cases**

|  | **Positive cases**  **(N=17)** | **Negative cases**  **(N=17)** | **Total**  **(N=34)** | **P=value** | **Odds ratio**  **(lower CI-upper CI)** |
| --- | --- | --- | --- | --- | --- |
| **Height SDS**^￥^  **(median [interquartile range])** | -2.87 [-4.10;-2.17] | -2.48 [-2.89;-2.39] | - | 0.335 | 2.82 (0.90-8.78) |
| **Facial dysmorphism^‡^** | 16 | 9 | 25 | 0.017 | 13.154 (1.397-666.676) |
| **Visual involvement** | 1 | 3 | 4 | 0.302 | 0.301 (0.005-4.259) |
| **ID/DD** | 12 | 14 | 26 | 0.688 | 0.524 (0.067-3.372) |
| **Microcephaly** | 9 | 11 | 20 | 0.728 | 0.623 (0.124-2.969) |
| **Abnormal finding in MR brain** | 2 | 3 | 5 | 0.631 | 0.631 (0.047-6.392) |
| **Skeletal anomalies** | 9 | 11 | 20 | 0.728 | 0.623 (0.124-2.969 |
| **Congenital heart disease** | 4 | 6 | 10 | 0.708 | 0.574 (0.093-3.170) |
| **Renal anomaly** | 3 | 5 | 8 | 0.688 | 0.524 (0.067-3.372) |

DD, developmental delay; ID, intellectual disability; MR, Magnetic Resonance; SDS, standard deviation score

￥ U-test was performed for continuous variables, whereas **^‡^**Fisher’s Exact test was performed for categorical variables.
