# Supplementary figures and images for: Clinical relevance of targeted exome sequencing in patients with rare syndromic short stature
Source: Orphanet J Rare Dis. 2021 Jul 3;16:297. doi: 10.1186/s13023-021-01937-8 (PMC8254301; doi:10.1186/s13023-021-01937-8)

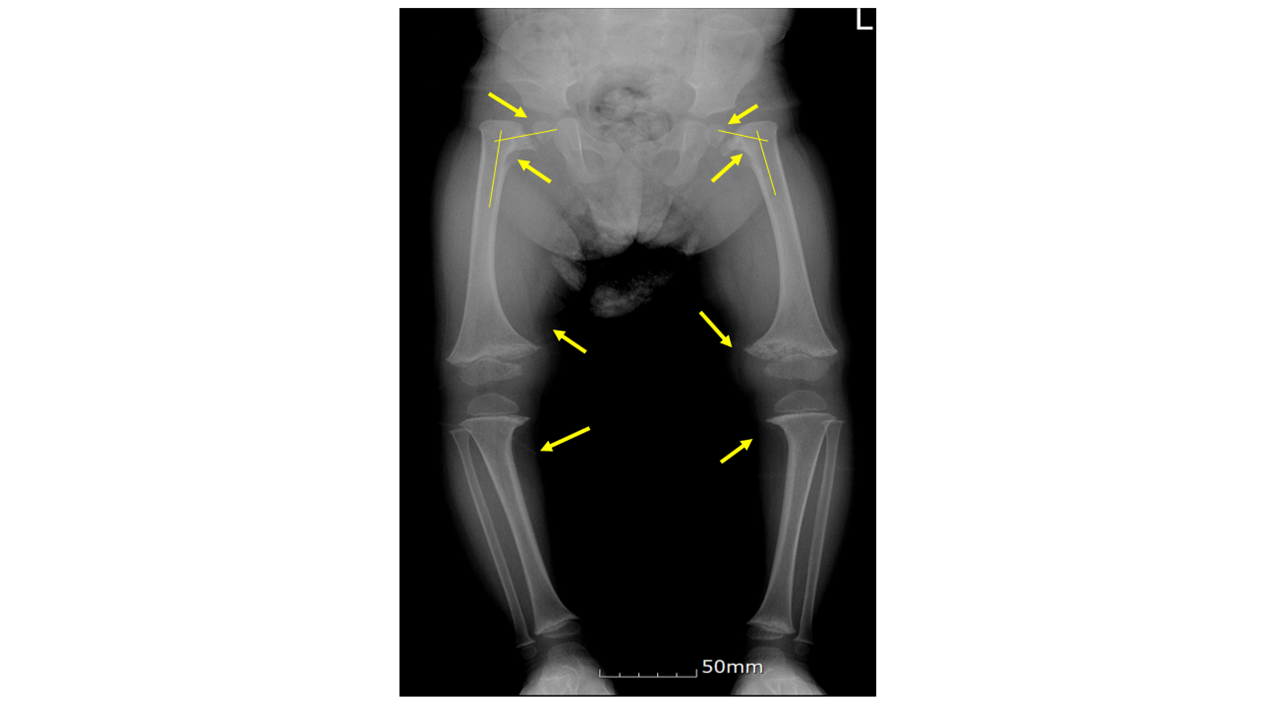

Supplement: Supplementary file 2 — Additional file 2. Radiographic image of patient K14 with microcephalic osteodysplastic primordial dwarfism (MOPD) type II. X-ray findings show coxa vara, genu varum, and both slipped capital femoral epiphysis marked by yellow arrows. [file 13023_2021_1937_MOESM2_ESM.tif]
